# Rehabilitation Supported by Immersive Virtual Reality for Adults With Communication Disorders: Semistructured Interviews and Usability Survey Study

**DOI:** 10.2196/46959

**Published:** 2023-10-31

**Authors:** Atiyeh Vaezipour, Danielle Aldridge, Sebastian Koenig, Clare Burns, Nilufar Baghaei, Deborah Theodoros, Trevor Russell

**Affiliations:** 1 RECOVER Injury Research Centre Faculty of Health and Behavioural Sciences The University of Queensland Brisbane Australia; 2 Katana Simulations Pty Ltd Adelaide Australia; 3 School of Health and Rehabilitation Sciences The University of Queensland Brisbane Australia; 4 School of Electrical Engineering and Computer Science The University of Queensland St Lucia Brisbane Australia

**Keywords:** communication disorders, speech and language therapy, rehabilitation, virtual reality, human-computer interaction, technology acceptance, acceptance, communication, therapy, usefulness, usability, survey, barrier, mobile phone

## Abstract

**Background:**

Individuals who have acquired communication disorders often struggle to transfer the skills they learn during therapy sessions to real-life situations. Immersive virtual reality (VR) technology has the potential to create realistic communication environments that can be used both in clinical settings and for practice at home by individuals with communication disorders.

**Objective:**

This research aims to enhance our understanding of the acceptance, usefulness, and usability of a VR application (SIM:Kitchen), designed for communication rehabilitation. Additionally, this research aims to identify the perceived barriers and benefits of using VR technology from the perspective of individuals with acquired communication disorders.

**Methods:**

Semistructured interviews and usability surveys were conducted with 10 individuals with acquired neurogenic communication disorders aged 46-81 (mean 58, SD 9.57) years after trialing an immersive VR application. The audio-recorded interviews were transcribed and analyzed to identify themes.

**Results:**

The quantitative data regarding the usability of the system associated with participants’ immersion experience in the VR application were promising. Findings from semistructured interviews are discussed across five key thematic areas including (1) participant’s attitude toward VR, (2) perceived usefulness of the VR system, (3) perceived ease of use of the VR system, (4) their willingness to continue using VR, and (5) the factors they perceived as challenges or facilitators to adopting this VR technology.

**Conclusions:**

Overall, participants in this study found the VR experience to be enjoyable and were impressed by the realism of the VR application designed for communication rehabilitation. This study highlighted personally relevant, immersive VR interventions with different levels of task difficulty that could enhance technology uptake in the context of communication rehabilitation. However, it is essential that VR hand controller technology is refined to be more naturalistic in movement and able to accommodate user capabilities.

## Introduction

Advancements in the capabilities and affordability of virtual reality (VR) technologies have contributed to the growing interest in the application of VR within health contexts [1-4]. In particular, the application of VR has the potential to facilitate clinical assessment and rehabilitation [5,6]. VR technology facilitates interaction with computer-generated, realistic images, sounds, and other sensations that simulate real-world environments. User interactions with VR systems may be “nonimmersive” involving a desktop, smartphone, or tablet screen displaying a digital world that can be explored, “semi-immersive” where partial immersion in the digital environment is made possible using projection screens (eg, driving or flight simulator), or “immersive” where the sense of physical presence or “being there” within the digital environment is facilitated through the use of head-mounted displays (eg, HTC Vive or Meta Quest) [7].

Studies suggest that individuals with conditions such as Parkinson disease, brain injury, acute and chronic pain, mild cognitive impairment, and posttraumatic stress disorder can benefit from the use of VR to improve physical skills [8-12], reduce pain [13-15], improve attention [16], and reduce anxiety [17-20]. In the discipline of speech-language pathology (SLP), research into the use of VR for the rehabilitation of communication disorders acquired during adulthood due to acquired brain injury (including traumatic brain injury), stroke, anoxia, brain infection, and diseases such as Parkinson disease and multiple sclerosis is emerging [21-23]. A small number of studies have shown positive effects on the functional (ie, real-life) communication skills of patients who had stroke associated with the delivery of communication rehabilitation via nonimmersive VR platforms such as digital worlds displayed on desktop computers [24]. A recent study using semi-immersive VR environments (eg, railway station, hotel, restaurant, supermarket, amusement park, and cinema) to deliver intensive treatment to individuals with poststroke communication impairment showed participants gained significant improvements in language-specific skills (eg, repetition and oral language comprehension), communication skills, and psychosocial well-being [4]. Interestingly, compared with a control group who received conventional treatment only (ie, individual communication therapy conducted in person), participants in this study who were exposed to the semi-immersive VR treatment made gains in a wider range of communication and language skill areas [4]. To the best of our knowledge, there have not been any studies investigating the use of immersive VR in the management of acquired communication disorders, although the potential usefulness of immersive VR has been recognized [22,25]. Immersive VR exposure therapy has been applied successfully to the treatment of neurologically intact individuals with public speaking anxiety [26]. Immersive VR has also been associated with reduced anxiety and improved confidence in public speaking environments for individuals who stutter [27-29].

The potential for immersive VR to create sufficiently realistic communication environments that could be accessed easily within the clinic and home practice setting is attractive for SLPs working with individuals with communication impairment [22,30,31]. In traditional clinic settings, it is challenging for SLPs to assist patients in transferring skills learned within the clinic into authentic, real-world communication environments [32]. Roleplay is often used as a bridge between clinic and real-world communication contexts. Given the complexity of communication and the influence of different environmental aspects (eg, noise and busyness) and personal factors (eg, emotions, motivation, fatigue, education, and culture) on communication success, it is useful for SLPs to work with patients on communication skills within a range of personally relevant environments. This often involves going out to a café, the hospital pharmacy, or some other relatively convenient setting where patients can practice interacting with others, applying their communication skills, and improving their confidence. However, these opportunities can be limited for clinicians constrained by time, workload, and service delivery protocols. Furthermore, these locations of convenience may not provide the personally relevant, contextualized, communication practice that is so critical to achieving optimal treatment outcomes [33]. VR can deliver a variety of realistic and immersive environments [23]. Moreover, VR environments may be manipulated by the clinician to increase or decrease complexity according to skill level, provide feedback on performance (visual, auditory, and haptic), and enable high repetition intensity to promote learning and improved participation in activities of daily living.

This research aimed to enhance our understanding of the acceptance, usefulness, and usability of an immersive VR application, SIM:Kitchen [31] designed for use in communication rehabilitation. Additionally, this study aimed to determine the perceived barriers and benefits to engagement with VR technology among people with neurogenic communication disorders. Identification of the determinants of VR acceptance by individuals with neurogenic communication disorders is an essential step prior to further development and refinement of the VR application for effective clinical uptake. This information will help not only to predict future adoption but also to develop appropriate solutions to address the potential barriers and challenges to the use of this VR technology.

## Methods

### Participants

Participants were recruited using a convenience sampling strategy from the general community in South East Queensland, Australia, via patient support groups, social media (ie, Facebook and Twitter), and snowball sampling methods. The University of Queensland’s media channels were also used to increase the exposure of the study. The study advertisements included a brief description of the study and a participant information sheet and explained the voluntary nature of participation in the research. Interested individuals were instructed to contact the research team by phone or email to assess their eligibility.

Eligibility to participate in the study required individuals to meet the following criteria determined by the expert judgment of a qualified speech pathologist on the research team (DA) to ensure their ability to communicate about their VR experience: (1) diagnosed with a neurogenic communication disorder (eg, aphasia, dysarthria, apraxia of speech, and cognitive-communication impairment) due to acquired brain injury (eg, stroke and traumatic brain injury), (2) the age of 18 years or older, (3) sufficient English language skills to understand and answer interview questions, (4) adequate cognition skills to support communication assessed via a brief cognition or memory screening test (moderate to mild cognitive impairment—score of 13 or above out of 30; Montreal Cognitive Assessment [34]), and (5) adequate mobility and balance to walk or maneuver their wheelchair with minimal assistance. A total of 10 individuals aged between 46 and 81 (mean 58, SD 9.57) years participated in this study with no dropouts. Most participants (n=7) were well-educated having completed at least undergraduate degree level study. Further details of participants’ demographic, diagnosis, and experience with technology are outlined in Table 1.

**Table 1:** Characteristics of individual participants’ demographics.

Participants	Age (years), gender	Highest education	Employment status	MoCA^a^	Diagnosis (year)	Communication disorder	Experience with technology^b^
P1	56, Female	Undergraduate	Not employed	21	Encephalitis (2016)	Mild dysarthria	4
P2	46, Male	High school	Not employed	16	Traumatic brain injury (2017)	Mild dysarthria, auditory memory difficulties	3
P3	48, Male	High school	Not employed	15	Stroke (2017)	Mild dysarthria, Mild to moderate auditory memory difficulties	2
P4	61, Male	Did not complete high school	Not employed	16	Hydrocephalus (1985)	Moderate dysarthria, mild to moderate cognitive-communication disorder, auditory memory difficulties	2
P5	69, Female	Postgraduate	Not employed	22	Stroke (2019)	Mild aphasia	4
P6	81, Male	Undergraduate	Retired	15	Stroke (2017)	Moderate aphasia (comprehension better than expression), auditory memory difficulties	4
P7	57, Male	Postgraduate	Full-time	21	Stroke (2021)	Mild aphasia, mild dyslexia (acquired), slight apraxia of speech	3
P8	50, Male	Undergraduate	Not employed	15	Stroke (2010)	Mild to moderate aphasia, auditory memory difficulties	2
P9	54, Female	Undergraduate	Not employed	20	Functional neurological disorder (2016)	Mild anomia	3
P10	60, Male	Postgraduate	Not employed	13	Stroke (2012)	Moderate aphasia (comprehension better than expression), auditory memory difficulties	3

^a^MoCA: Montreal Cognitive Assessment.

^b^Experience with technology scale (1=inexperienced to 5=experienced).

### Ethical Considerations

This study was conducted according to the ethical guidelines of the University of Queensland and the National Statement on Ethical Conduct in Human Research (approval 2019001282). Prior to inclusion in the research, all participants were fully briefed, both orally and through the written participant information sheet, and written informed consent was obtained. All the data collected in this study were deidentified to ensure the privacy and confidentiality of participants’ data. After the completion of the study, participants received a US $77 gift voucher as a token of appreciation for their participation.

### VR Platform, Hardware, and Description

The immersive VR application evaluated in this research was designed and developed following the principles of the human-centered design approach to ensure it meets the needs of end users [35]. The iterative design process involved a series of interviews with SLP as an initial step to enhance safety prior to the involvement of patients [31].

**Figure 1:**
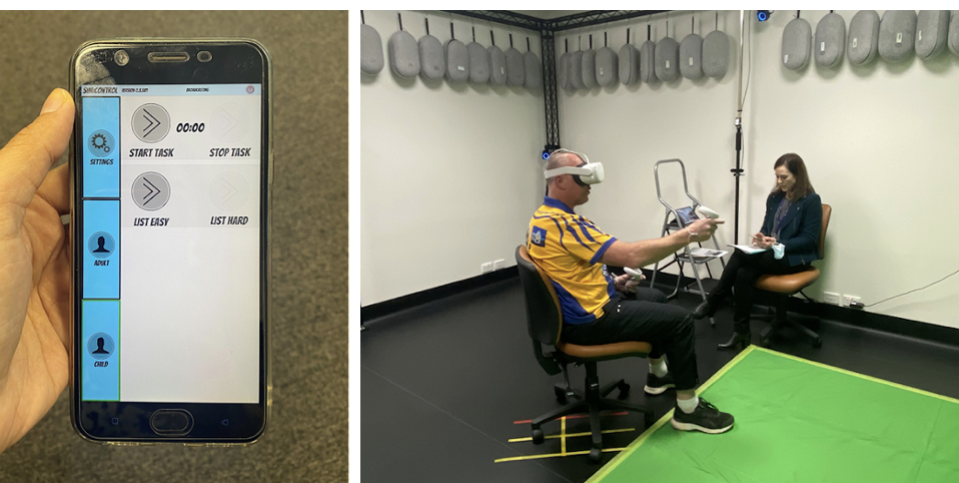
Speech pathologists can manipulate the interactions in the virtual reality scenario via a tablet or mobile phone (left) and patients wear a Quest headset and use 2 hand controllers to interact with objects (right).

**Figure 2:**
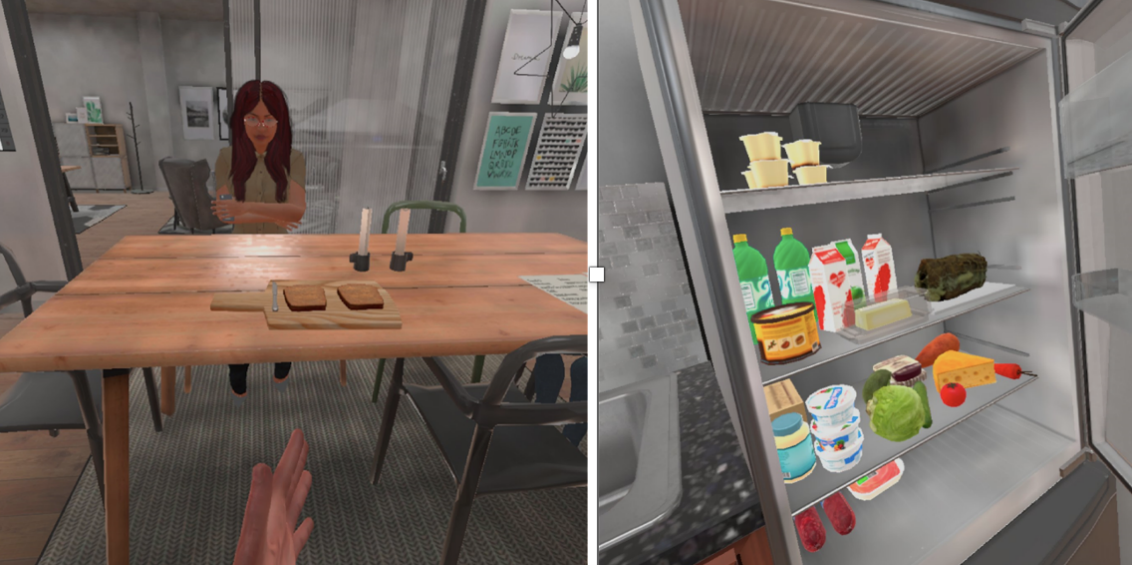
A kitchen and adult avatar in a home (left) and objects in the fridge that could be interacted with (right).

The VR application consisted of a simulated VR kitchen environment (SIM:Kitchen) developed using Unity Technologies game engine (version 2020.2.3), and ran on a Meta Quest 2 headset and hand controllers in Room scale mode in either seated or standing position. User interactions were implemented using HurricaneVR’s physics interaction toolkit (Cloudwalkin Games). The simulated VR kitchen enables two types of user interactions. These are (1) the SLP communicating with the participants via an adult avatar to perform a series of tasks in the SIM:Kitchen environment (eg, participant has to describe the room or objects or sounds around them, have a conversation with an adult within the room, introduce themselves to a child within the room, and have a conversation with the child, request something to drink, and make a sandwich). The SLP can also enable lip-syncing of the adult and child characters via an Android app that wirelessly connects to and controls the SIM:Kitchen application and (2) the participants can remain seated and safely interact with objects in the VR kitchen using the hand controllers while wearing a Meta Quest headset where they can hear and respond to communications and instructions delivered by the SLP. Figures 1 and 2 show the study setting and screenshots of the VR application.

### Data Collection Procedures and Measures

Participants attended a 90-minute session at RECOVER Injury Research Centre, The University of Queensland. Upon arrival, participants completed a brief questionnaire regarding demographics and level of experience with using technology. Thereafter, a researcher (AV) provided detailed information about the functionality of the VR headset and hand controllers. Participants were given approximately 10 minutes to familiarize themselves with the VR equipment to ensure they felt comfortable using the hand controllers and interacting with digital objects within the simulated VR kitchen environment. Participants remained seated in a chair throughout the introductory task to minimize the risk of disorientation or fall due to the immersive nature of the VR application.

Next, several communication tasks were completed within the simulated kitchen environment, of approximately 20 minutes’ duration. An experienced speech pathologist on the research team (DA) instructed participants to perform a series of tasks and interactions with other avatars present in the SIM:Kitchen environment. All participants completed a similar list of tasks that included describing the room or objects or sounds around them, having a conversation with an adult avatar within the room, introducing themselves to a child avatar within the room, having a conversation with the child, and making the child a sandwich. Participants were given short breaks (approximately 5 min) between each task to ensure their comfort and check for any symptoms of motion sickness while wearing the VR headset.

Following the VR trial, participants completed a series of questionnaires and participated in a short interview conducted by a member of the research team (DA). Three questionnaires were completed by each participant (duration approximately 10 min) with the assistance of the same researcher (DA) to ensure comprehension of the content and answer questions as required. Questionnaires included the following:

1. System Usability Scale (SUS) [36,37], which included 10 items (5-point Likert scale, “strongly disagree” to “strongly agree”) generating composite scores ranging from 0 to 100 to evaluate the usability of the VR system.
2. Subjective mental workload (NASA Task Load Index, NASA-TLX) [38], which is designed to gauge the workload and effort participants put into the tasks including mental demand, physical demand, temporal demand, performance, effort, and frustration while engaging in the VR experience. NASA-TLX included 6 items. Each item is scored on a 0-100 scale with scores across all 6 items summed and then averaged.
3. Simulator Sickness Questionnaire (SSQ) [39], which included 16 items (4-point Likert scale, “not at all” to “severe”) to ascertain comfort levels and identify any adverse impacts of the VR experience. Final scores were calculated as per instructions provided by Kennedy et al [39].

Finally, a semistructured interview (approximately 30 min) was conducted to explore participants’ views about their experience of the SIM:Kitchen to determine perceived usability and acceptance of VR technology in general and identify benefits and barriers to potential use within speech pathology sessions. The interviewer (DA) did not have a prior relationship with the participants. An interview guide was developed by AV and reviewed by other members of the research team (DA and DT). The interview guide (Multimedia Appendix 1) was developed following the theoretical framework of the technology acceptance model (TAM) [40,41] due to its demonstrated ability to capture the concepts of perceived usefulness, usability, ease of use, and intention to use as measures of user acceptance. The TAM has been validated in a range of studies of user acceptance in public health [42]. Moreover, replication studies have suggested that the TAM is a valid and robust model [43,44]. In this research, quantitative surveys were used to assess the usability of the system. Subsequently, semistructured interviews were conducted to provide deeper insights into the quantitative findings. This approach aimed to enhance the comprehensibility of the results and to gain insights into the factors that hinder, or aid communication rehabilitation facilitated by an immersive VR application.

### Data Analysis

The data from questionnaires (ie, demographic and postquestionnaires) were analyzed descriptively using Excel (Microsoft Corp; AV). The audio-recorded interviews were transcribed (AV and DA) and imported into NVivo (version 11; QSR International). To reduce the participant’s time commitment, transcripts were not returned to the participant for comment. However, participants were encouraged to contact the research team if they wished to share any additional information after the interview. A qualitative content analysis was conducted by 2 researchers (AV and DA) with experience in qualitative data analysis to identify patterns across responses and their relation to research questions [45]. Initially, data were categorized based on the principles of the TAM. Themes that did not fit within this framework were considered during the coding process. This review and refinement process led to the development of the final themes and subthemes. Two other researchers (CB and NB) also reviewed the themes and subthemes to validate the themes and enhance the trustworthiness of the coding. Discrepancies were discussed and guided the thematic development, resulting in coherent themes reflecting a comprehensive and precise set of meanings for participant comments. The Research Team and Reflexivity Statement are included in Multimedia Appendix 2. The Consolidated Criteria for Reporting Qualitative Research (COREQ) checklist was used to guide the qualitative reporting [46].

## Results

### Quantitative (System Usability, Workload, and Motion Sickness)

The average System Usability Scale score of the SIM:Kitchen application was 60.75 out of 100 suggesting an average usability [37] from the perspective of participants. Additionally, the average scores of NASA-TLX workload across mental (mean 39, SD 34.7), physical (mean 34.50, SD 30.9), temporal (mean 12.5, SD 15.3), performance (mean 39.50, SD 23.9), effort (mean 41, SD 33.2), frustration (mean 26.25, SD 23.5), and total workload (mean 33.3) show low levels of workload associated with using the SIM:Kitchen application. Finally, the average Simulator Sickness Questionnaire scores for nausea, oculomotor, and disorientation and total score were 14.79, 18.27, 17.82, and 18.1, respectively, suggesting low motion sickness symptoms after trialing the VR system [47].

### Qualitative (Semistructured Interview)

#### Overview

The content of the semistructured interviews conducted for this study can be organized into five key thematic areas including (1) attitude toward VR and SIM:Kitchen, (2) perceived usefulness of VR system, (3) perceived ease of use of VR system, (4) intention to use VR, and (5) perceived adoption barriers and enablers. These key themes with underlying subthemes or categories are presented in narrative form and summarized in Textbox 1.

**Summary of themes and subthemes.**

Attitude to virtual reality (VR) and SIM:Kitchen

- Enjoyable and engaging
- Without real-world distractions
- Representative of real life

Perceived usefulness of VR system

- Potential benefits of VR in communication rehabilitation
- Potential benefits from provision of performance feedback
- Potential benefits from design of personalized, goal-based tasks with variable levels of difficulty
- Potential benefits from design for use with groups or remotely
- Potential benefits of alternative VR scenes or scenarios

Perceived ease of use of VR system

- Physicality and interaction with VR equipment
- Experience of immersive VR environment

Intention and motivation to use VR

- Provision of instruction or support
- Feedback on performance
- Demonstrated benefits

Perceived adoption barriers or enablers

- Personal factors
- User-friendliness
- Accessibility to the range of clients or communication needs
- Demonstrated benefit

#### Attitude Toward VR and SIM:Kitchen

Overall, participants in this study were positive about their experience of the immersive SIM:Kitchen environment and the possibility of using VR for communication rehabilitation. Participants commented that the SIM:Kitchen environment was “wonderful” (P6) and “a great idea” (P2 and P7) that “could be really beneficial for (communication) practice” (P5). In addition, participants felt that the experience of being in the SIM:Kitchen environment was “engaging” (eg, P9), “not boring” (eg, P2), and rather “enjoyable” (eg, P3). One participant stated they were “blown away” by the simplicity, realism, and ease of use of the VR system (P9). Another commented that having a window you could see out of in the SIM:Kitchen environment was a nice feature, especially for people prone to claustrophobia (P7). It was apparent to some participants that VR could transport you into a scenario that could be enacted rather than “reading stuff off a sheet of paper” (P5), as may be the case in traditional communication rehabilitation activities. One participant observed that for her the SIM:Kitchen environment created a “clean space” without real-world distractions where “you can leave your pre-existing anxieties about ... speech behind” [P7]. Having the visual cues from the SIM:Kitchen environment available to support communication was considered helpful and representative of real-life communication situations (eg, P2 and P10). However, for some, VR was still considered to be limiting and a “barrier to reality” compared with practicing communication within real-life situations (P4 and P7).

#### Perceived Usefulness of VR System

##### Benefits of VR in Communication Rehabilitation

A number of participants expressed uncertainty about how VR could be used to assist in the rehabilitation of communication following acquired brain injury (P3, P4, and P9). One participant in the study initially expressed strong negativity regarding the usefulness of VR for communication rehabilitation stating that “people would like the idea of VR” but that dealing with the frustration of using VR combined with the existing frustration of having aphasia and trying to communicate “... they’re going to feel like a total failure” [P4]. However, as the interview progressed and ideas were explored, this participant’s stance about the usefulness of VR for communication rehabilitation shifted as she suggested VR may be useful for building communication skills with people who enjoy gaming, especially 20- to 25-year-olds who may already have gaming skills and feel comfortable with VR.

The majority of participants were positive about the potential usefulness of VR for communication therapy “Having practice speaking in a safe environment would be very helpful” [P2], “... you put yourself in the (VR) scenario (to practice communication). I think this (VR) would be much more engaging. A lot of people would really enjoy that a lot more ...” [P5]. The concept that VR might remove the fear of failure and help develop communication confidence was discussed by participants in the interview (eg, P1, P2, P4, and P7). It was also suggested that VR could be particularly useful in the early stages following a stroke, where a 3D VR environment could provide realistic visual cues to assist in improving word-finding skills (P4, P5, and P10). One participant (P5) generated numerous ideas about how VR could be useful for communication rehabilitation:

... at the very beginning in rehab, when I couldn’t speak very well and coming up with certain words ... of things (that flashed up on an iPad) and you had three seconds to name what the thing was ... you could do the same thing in the grocery store (VR environment) ... things could be going by and you have to say ‘broccoli, cucumber, onion’ ... I think it would really help

and

... when I was just leaving rehab they wanted me to read more details, longer things and do something like prepare a recipe. So that's something I think (you could do) in the (VR) kitchen ... Break up these two eggs and have your mixing bowl and just start doing something like that and see if the person can follow through all the things like that (steps) and prepare something.

##### Design Features to Maximize Usefulness

Design features suggested for future development of VR communication rehabilitation systems included personalizing goal-based tasks with different levels of difficulty (eg, P2). In addition, the capacity of the VR system to offer performance ratings and feedback options was identified as an essential feature to assist with communication rehabilitation: “I always like to know if I’ve done something to improve” [P2]. The potential for VR to be used with groups of individuals undertaking communication rehabilitation was also considered, especially in relation to remote connection and conversation with others that could bring enjoyment and build confidence (eg, P1, P2, and P9). Participants commented that there would be definite benefits in creating VR environments, where multiple users could log in from different locations to participate in the conversation (eg, P9).

##### Benefits of Alternative Scenes or Scenarios or Tasks

When asked to consider other environments that would be useful to simulate using VR to assist with communication rehabilitation, 1 participant answered: “Any environment would be good ... as long as there was somebody you could talk to” [P1]. Other participants liked the idea of a supermarket: “... where you have your list of things and an empty basket and then have to go and look up and down the aisle to find items to put in the basket” [P5], where opportunities for practicing simple small talk could be created (eg, while standing in the queue at the deli or checkout; P2). Another participant suggested he would be more inclined to communicate within a VR café scenario or men’s shed environment than a kitchen (P9). Additional environments mentioned as potentially useful for VR communication rehabilitation were a doctor’s surgery, a shopping center, communication with neighbors when taking the dog for a walk, and a pharmacy (eg, P1, P2, P9, and P10).

#### Perceived Ease of Use of the VR System

##### Physicality and Interaction With Hardware

Moving things with the hand controllers was frustrating, not realistic, and somewhat problematic for many participants (eg, P1, P2, P3, P4, and P6). One participant (P6) felt that for him, activating a different button on the hand controllers might have assisted him to use them more successfully. Others felt that with additional time and practice, the use of hand controllers might become easier and less problematic for some people but may still be problematic for individuals with weakness or paralysis of their upper limbs (P2). One participant felt that the operation of the VR equipment was too complicated for individuals already struggling to focus on their communication (P4).

General mobility and movement within the VR environment felt restricted for some participants (eg, P10) due to the safety requirement of the study for participants to remain seated rather than stand up and move around to explore the simulated kitchen environment. Others felt comfortable and safe seated on a swivel chair during their immersion in the VR environment (eg, P7). For a small number of participants, vision through the headset was blurry at times, requiring adjustment (P2 and P10). The VR headset itself was not considered to be bothersome or uncomfortable for most participants. However, it was acknowledged that for some people the headset may be considered heavy or uncomfortable on the face (P3 and P4).

##### Experience of the Immersive VR Environment

The immersive VR environment was considered “not real but ... life like” [P7]. All participants in the study felt comfortable within the SIM:Kitchen environment. Only 1 or 2 participants mentioned feeling slightly disorientated at the outset or end of the immersive VR experience (eg, P5 and P8). It was supposed that some individuals may require more time than others to acclimatize to immersion in VR environments (P8). The Meta Quest equipment used in this study did not allow for sound delivery through the headset, which diminished the sense of immersion and engagement for some participants (eg, P5).

#### Intention and Motivation to Use VR

Participants indicated that they would be motivated to use VR if training to use the VR system was provided and if performance feedback in the VR environment could be achieved (eg, P1, P2, P3, and P9). “I think if you have direction and feedback, and it was showing you were improving (that would motivate me to use the VR system)” [P2]. For most participants, the intention to use VR for communication rehabilitation was very much outcome-dependent based on the demonstrated benefits of VR to assist with communication improvement. “If someone could show me that it would be useful ... I will jump straight into it” [P9]. Some suggested that they would also be open to using VR for home practice (eg, P3 and P6) or as a “check-in tool” or reminder to implement speech strategies (P2). However, the assistance and supervision by a speech pathologist to manage the implementation of immersive VR for communication rehabilitation were considered essential for some (eg, P2).

#### Perceived Adoption Barriers and Enablers

##### Personal Factors

A number of factors were considered to have potential impacts on the adoption and continued use of VR for communication rehabilitation. For instance, a tendency to become claustrophobic could impact the use of VR:

People who are claustrophobic probably wouldn’t like it. It doesn’t sort of worry me ‘cause I can resign myself to the fact that I’m going to use it so I do it. But people... they mightn’t like that (VR) because they mightn’t like that (headset) on their face.

P1

In addition, it was suggested that immersion in the 3D VR environment and learning to use the hand controllers could take some people more time than others, which could affect the successful adoption of VR (P2 and P8). Moreover, individuals who feel dizzy or otherwise uncomfortable within the immersive VR environment may be reluctant to pursue VR as a means to improve their communication (P2). Personal drive to improve was also mentioned as a factor that could influence the adoption of VR for communication rehabilitation among individuals with acquired communication disorders (P1 and P4).

Physical ability, level of cognitive and communication impairment, as well as the level of confidence in communicating in the real world were raised as additional factors that could impact on the adoption of immersive VR for the rehabilitation of individuals with acquired communication disorders (eg, P2, P4, and P7). In relation to communication confidence, individuals who already felt confident to practice their communication in real-world situations did not think that they would use VR (P4 and P7).

##### User-Friendliness

Technical difficulties and the need for assistance to operate the VR system were identified as factors that could limit the adoption of VR for communication rehabilitation. “When technology doesn’t work you get frustrated and give up” [P2]. Frustration with the inconsistency of operation of the hand controllers to interact with items in the SIM:Kitchen environment was identified as a factor that may negatively impact upon adoption and continued use (eg, P3)

While headset comfort was not highlighted as a specific issue for participants in this study, it was acknowledged that VR headset comfort could impact upon adoption and continued use of VR for some people (eg, P1, P3, and P8). Clarity of vision through the headset was raised as a minor issue for several participants and a factor that could discourage the use of VR (eg, P1, P2, and P10).

##### Accessibility and Demonstrated Benefit

Participants in this study considered that the successful adoption of VR among individuals with communication disorders would rely foremost on demonstrated benefits (eg, P1 and P9). Moreover, participants felt that motivation for continued use of VR would occur if feedback while using the device showed improvement in communication outcomes for individuals (eg, P2 and P3).

The availability of VR equipment at an easily accessible facility or within the user’s home was noted as a potential enabler to the adoption of VR for communication rehabilitation. Conversely, increased travel time to access VR equipment for communication rehabilitation was considered a potential deterrent to use and a barrier to adoption (eg, P1). Physical and communication abilities were also considered factors that could variably influence individuals’ access to and willingness to adopt VR for communication rehabilitation. “There’s a wide range of disability (in terms of) limb function ... that’s part of it (to consider)” [P5].

## Discussion

### Principal Findings

This study compliments and extends upon our earlier mixed methods study exploring the views of speech-language pathologists about the potential usefulness of immersive VR for communication rehabilitation [31]. A mixed methods approach was used to explore the perspectives of individuals with neurogenic communication disorder as to the perceived acceptance, usefulness, and usability of immersive VR within the context of communication rehabilitation. Quantitative measures of system usability, mental workload, and motion sickness associated with participants’ immersion experience in SIM:Kitchen were promising. Despite being a research prototype, the SIM:Kitchen VR application was considered to have average usability according to participants in this study. In addition, participants were not overly mentally taxed when using SIM:Kitchen and experienced low levels of motion sickness symptoms, if any, while using the VR system.

Key findings from the semistructured interviews conducted with participants in this study are discussed later with particular attention to the potential usefulness as well as identified barriers and enablers to the future adoption of VR for communication rehabilitation. Of the 10 participants in this study, all participants were selected based on their ability to communicate sufficiently well to offer their opinions about the immersive VR experience. While age, level of education, cognitive ability, level of communication impairment, and degree of prior exposure to or experience with technology may have affected participants’ generation of ideas about the potential use of VR for communication rehabilitation, general optimism toward VR was clear from responses. Overall, participants found the VR experience to be enjoyable and were impressed by the simplicity and realism of the SIM:Kitchen environment. Similar positive attitudes are reflected in other studies that have used VR environments with health care students, workers, and patients [31,48,49].

It is well known that a positive perception of the usefulness of a particular technology to address a specific need is critical to the successful adoption of that technology [40,50]. Some participants in this study proposed specific communication rehabilitation tasks that could be carried out via VR to enhance therapy engagement and outcomes, suggesting an openness to VR as a therapy tool. In addition, participants put forward ideas for alternative VR scenes or scenarios (eg, café, doctor’s surgery, and pharmacy) that could provide useful contexts for functional communication therapy tasks. Personally relevant interventions grounded in ecologically valid, real-life contexts are essential to maximize communication rehabilitation outcomes [33]. However, exposure to a wide range of personally relevant communication environments during traditional clinic-based rehabilitation is often difficult to achieve.

The potential of VR to create valid contexts for communication skills practice and the development of communication confidence has long been recognized [51]. Participants in this study could envisage the use of immersive VR in creating realistic representations of real-life communication environments. Moreover, it was considered that these simulated VR environments could hold additional value through the provision of less complex, less distracting, and “safer,” less threatening, contexts within which to practice communication skills and gain confidence. This feature of VR to reduce or build up complexity may be important given the often-reduced ability following brain injury to inhibit environmental distractions (eg, background noise and visual distractors) and encourage attention on specific elements [52]. Confidence to communicate was a key challenge acknowledged by all participants in this study. The safe and supportive environment offered by immersive VR could help to facilitate the development of communication confidence.

A recent study reporting on the outcomes of conversational therapy for individuals with aphasia delivered through semi-immersive VR scenarios representative of everyday life (eg, supermarket, restaurant, amusement park, railway station, and post office) showed benefits for communication functioning including oral comprehension, repetition, and written language, as well as psychological aspects such as self-esteem and mood state [4]. Other studies delivering intervention via VR platforms have also shown improved communication outcomes for this group measured by formal functional communication assessment and participants’ reports of maintained communicative confidence up to 1-year posttreatment [24,53,54]. Furthermore, positive effects on the generalization of functional communication skills from the digital environment to the real world have been demonstrated [21]. For participants in this study, these improvements in communication were considered essential and firmly linked to motivation and intention to use immersive VR applications designed for communication rehabilitation.

Enjoyment was another factor participants touched upon as motivation for the use of immersive VR applications in communication rehabilitation. Studies using digital gaming therapies have reported therapeutic enjoyment to be positively correlated with clinical improvements [53,55,56].

Balancing the level of difficulty of tasks (including task instructions) and an individual’s abilities may also be important in optimizing enjoyment and motivation to use VR [57]. It is conceivable that the inclusion of gamified VR tasks within communication rehabilitation programs that increase therapeutic engagement and enjoyment could foster enhanced outcomes. Moreover, manipulation of task complexity, measurement of performance, and feedback on performance (proposed determinants of adoption of VR for participants in this study) could also be incorporated into gamified VR communication practice tasks. Expert feedback on performance is essential for improving skills during speech pathology sessions. The question of whether similar benefits could be achieved with feedback delivered via immersive VR applications is an interesting one since the feedback has many different modalities (eg, qualitative or quantitative, specific or global, and implicit or explicit) and targets (eg, speech articulation clarity or loudness and overall communication success) and should be delivered with varying timing and frequency depending on individual ability, progress, and the goals of treatment [58]. Ideally, future design and development of immersive VR technology for use in communication rehabilitation should address the user-friendly and well-timed delivery of these distinct types of feedback. Addressing such complexities well may impact significantly upon the successful adoption of the technology within the health rehabilitation setting [59].

While the perceived usefulness of technology is highly important, successful adoption of technology is also intrinsically linked to ease of use and available support [40,60]. Mastering the use of the hand controllers was possibly the most difficult aspect of the immersive SIM:Kitchen experience for participants in our study. For many, the distraction and frustration created by difficulty using the hand controllers diminished realism and sense of immersion and detracted from the communication tasks within the VR experience. There are many occasions where communication and physical actions are carried out together (dual tasking, eg, having a conversation with another person while preparing a sandwich). In addition, hand gestures may be used by some individuals to facilitate and augment their communication. Therefore, for the purposes of communication rehabilitation, it is essential that VR hand controller technology is refined to be more naturalistic in movement and able to accommodate individual capabilities (eg, weakness, lack of dexterity, and precision of movement). Future steps in the SIM:Kitchen project will encompass enhancing the functionality of VR hand controllers through an iterative development process, which will be coupled with extensive testing involving patients. Additionally, we will explore the potential integration of hand gestures to facilitate a more naturalistic interaction, aiming to further enhance the SIM:Kitchen usability. Participants also felt that the sense of realism and immersion within the VR environment would have been enhanced by delivering high-quality speech through the headset. Enhancing the gestures (especially lip movement during speech), facial expressions, and responsiveness of the characters could also improve the realism, immersion, and authenticity of communication interactions within the VR environment. Congruent cues such as these, delivered across multiple senses (ie, auditory, visual, olfactory, and tactile) are known to enhance immersion and sense of presence within VR environments [61]. Moreover, the inclusion of these cues, especially high-quality audio, may be important for optimizing memory and learning within the context of rehabilitation using immersive VR [62].

Ease of access to the VR system was highlighted as an important determinant of intention to use the device for communication rehabilitation. For participants in this study, direction from a clinician, and continuing support from the SLP about how to use the VR system and engage within the SIM-Kitchen environment to complete the directed tasks, was important. Similar views were held by participants in a recent study of aphasia intervention delivered via a computer-based digital environment, where the relationship with the person who delivered the intervention was highly valued [53]. However, from the clinician’s perspective, the opportunity for client users to access and use VR asynchronously, without direct clinician supervision, is attractive [31,60]. Access to home-based rehabilitation options is convenient and could enable increased practice intensity for enhanced recovery of function [63]. With practice, client users could develop the skill and confidence to use VR systems and applications more independently at home or with the assistance of a friend, relative, or support worker rather than relying solely on the clinician. As VR technology develops and becomes simpler and safer to use, it is easy to imagine greater use of this technology in the future for home-based rehabilitation.

### Study Limitations and Recommendations for Future Research

Despite every attempt to recruit a representative and larger sample, the number of participants was small in this study (N=10). However, the results mirror those of other recent studies suggesting that carefully designed immersive VR may be a beneficial inclusion in communication assessment and rehabilitation [31,64]. There was a relatively high median age range (mean 58, SD 9.57 years) of participants in our study that may have limited the breadth of information gathered. The notion that younger individuals may have more experience with immersive VR technology and a greater potential for acceptance of the technology for rehabilitation should be examined. Only 2 participants (P9 and P10) in our study reported some minimal experience with the use of immersive VR. Future studies should include larger and more representative participant groups, incorporating a wider range of ages and demographics, encompassing individuals with varying levels of technological familiarity, diverse communication and neurological impairments, and differing countries of origin and cultures.

While the participant group for this study was relatively well-educated and did not include individuals with more severe cognitive and communication impairments, this was considered reasonable given the current lack of knowledge about the safety and suitability of immersive VR for individuals with acquired brain injuries.

The recent impacts of COVID-19 worldwide have expanded the development and adoption of technology including telehealth, digital care, artificial intelligence, and robotics [65]. Moreover, a return to pre–COVID-19 levels of engagement with technology seems unlikely. The time is ripe to take advantage of heightened awareness and openness to technology to explore opportunities and challenges associated with the use of immersive VR for communication rehabilitation. Future efforts to design immersive VR for communication rehabilitation should consider personalized VR intervention with different levels of interactivity and realism, taking into account individual abilities and level of physical or cognitive or communication impairment. Safety aspects should be prioritized while enabling adjustment of complexity of tasks, provision of feedback on performance, and achievement of outcomes that are at least equivalent to those obtained through traditional communication therapy approaches. The potential use of hands as opposed to the VR controllers and remote options for the use of VR technology via telehealth for communication rehabilitation should also be explored.

### Conclusions

The results of this usability study were positive toward the use of immersive VR for communication assessment and rehabilitation and highlighted the importance of an iterative, co-design process involving end users in designing and developing this technology to maximize engagement and benefits. Additionally, this study revealed personally relevant, immersive VR interventions with different levels of task difficulty are required in the context of communication rehabilitation. However, VR hand controller technology needs to be optimized for more naturalistic movement and accommodation of differences in physical capabilities (eg, hand weakness and mobility impairment).

## Appendix

Multimedia Appendix 1 Interview guide.

Multimedia Appendix 2 Research Team and Reflexivity Statement.

## Abbreviations

COREQ: Consolidated Criteria for Reporting Qualitative Research
NASA-TLX: NASA Task Load Index
SLP: speech-language pathology
SSQ: Simulator Sickness Questionnaire
SUS: System Usability Scale
TAM: technology acceptance model
VR: virtual reality

## Data Availability

The data collected from the participants in this research are not publicly available in order to comply with the ethics approval of the study.
