# Supplementary material for: Rehabilitation Supported by Immersive Virtual Reality for Adults With Communication Disorders: Semistructured Interviews and Usability Survey Study
Source: JMIR Rehabil Assist Technol. 2023 Oct 31;10:e46959. doi: 10.2196/46959 (PMC10646677; doi:10.2196/46959)
Supplement: Multimedia Appendix 1 [file rehab_v10i1e46959_app1.docx]

**Multimedia Appendix 1.** Interview guide.

The purpose of this study is to find out from a sample of people who have communication difficulties resulting from neurological impairment/disease, their views about the virtual reality system that you have just experienced. We also want to know their views about VR (virtual reality) more generally. Now I would like to ask you some questions about your own experience of the VR system. Before I begin, do you have any questions?

**QUESTIONS**

**Q1.** Overall, what are your first impressions of the SIM:Kitchen VR system you have just experienced? What did you like/not like about it?

**Q2.** If VR was available to be used for your speech/communication rehabilitation, how could you see VR being used? Do you think VR could assist you to develop your communication skills? How?

**Q3.** How willing would you be/have been to have VR tasks similar to the SIM:Kitchen tasks included in your own speech pathology program? Under what conditions? In what way(s) would you find VR beneficial/not helpful?

**Q4.** Can you think of any potential problems/barriers to using the system? discomfort/inconvenience using the VR equipment? What might make you want to stop using the system?

**Q5.** Can you think of any ways we could improve the simulated kitchen or the VR system in general?

**Q6.** Can you think of a specific context (e.g., real-world environment) that could potentially be simulated in a VR environment for your speech therapy? How would it be used?

**Q7.** Is there anything else you would like to add? Are there any questions you would like to ask?
