# Supplementary material for: Rehabilitation Supported by Immersive Virtual Reality for Adults With Communication Disorders: Semistructured Interviews and Usability Survey Study
Source: JMIR Rehabil Assist Technol. 2023 Oct 31;10:e46959. doi: 10.2196/46959 (PMC10646677; doi:10.2196/46959)
Supplement: Multimedia Appendix 2 [file rehab_v10i1e46959_app2.docx]

**Multimedia Appendix 2.** Research Team and Reflexivity Statement.

Dr. Atiyeh Vaezipour (AV) and Ms Danielle Aldridge (DA), were both female academic researchers at the time of the study. AV is a research fellow with the PhD in human-computer interaction. DA is a qualified speech-language pathologist with over 14 years of clinical and academic experience. DA had previous experience in designing, developing and implementing simulation courses (not involving VR) with speech-language pathology students. DA conducted the interviews while AV was taking field notes and supporting the VR technology set-up. DA are AV are experienced qualitative researchers with a successful record of previous qualitative research publications. AV and DA did not have any previous relationship with participants. The reason for conducting the research shared with participants was our interest in finding potential benefits and challenges for implantation of immersive VR technologies for communication rehabilitation.

Dr. Koenig is an experimental psychologist with a PhD in Human Interface Technology. He was involved in the development of the SIMKitchen application and due to his financial interest in the evaluated VR application, he was not involved in the data collection and analysis of the data. He has provided the technical expertise in relation to VR application and reviewed the manuscript.

Dr. Clare Burns (CB) is an advanced speech pathologist (PhD) and clinician research fellow at Royal Brisbane and Women's Hospital, Queensland, and an honorary senior lecturer at The University of Queensland, Australia (UQ). She has more than 20 years of clinical experience and, has conducted research in speech pathology and technology-enabled healthcare. She oversaw the study and reviewed qualitative analysis and manuscript.

Dr. Nilufar Baghaei (NB), is a Senior Lecturer (PhD) within the School of Electrical Engineering and Computer Science at The University of Queensland and the Co-Director of the Extended Reality Lab. She has expertise in Virtual/Augmented Reality in Health/Education and Persuasive Technology. She oversaw the study and reviewed qualitative analysis and manuscript.

Professor Deborah Theodoros (DT) is a distinguished emeritus professor, PhD in speech-language pathology with expertise in the field of telerehabilitation and the treatment of speech disorders. She was involved in the design of this study and oversaw the study and reviewed the manuscript.

Professor Trevor Russell (TR) is a Professor in the Division of Physiotherapy within the School of Health and Rehabilitation Sciences at the University of Queensland. He has a PhD in Telerehabilitation and is director of RECOVER Injury Research Centre at the University of Queensland. He oversaw the study and reviewed the manuscript.
